# Renal clear cell carcinoma metastasis to the breast ten years after nephrectomy: a case report and literature review

**DOI:** 10.1186/s13000-017-0666-8

**Published:** 2017-11-02

**Authors:** Yanjun Xu, Rui Hou, Qijie Lu, Yifan Deng, Bin Hu

**Affiliations:** Department of Ultrasound in Medicine, Shanghai Institute of Ultrasound in Medicine, Shanghai Jiao Tong University Affiliated Sixth People’s Hospital, 600th Yishan Road, Xuhui District, Shanghai, 200233 People’s Republic of China

**Keywords:** Renal cell carcinoma, Neoplasm, Metastasis, Breast, Mammography, Ultrasonography

## Abstract

**Background:**

Renal cell carcinoma most commonly metastasizes to the lungs, skeleton or liver. Metastatic renal cell carcinoma to the breast is very rare, especially for clear cell carcinoma, and few cases regarding this condition have been reported.

**Case Presentation:**

The case we presented was a 68-year-old Chinese female with metastatic renal clear cell carcinoma of the left breast 10 years after a nephrectomy. Identification of the metastatic renal clear cell carcinoma in the breast required multiple breast imaging modalities. Imaging showed a single, ovary-shaped, well-defined hypo-echoic mass, with abundant blood flow on ultrasound images. The mass was enhanced early on MRI, and it was hypointense on a T1-weighted image and hyperintense on a fat-saturated T2-weighted image. Following surgical excision of the tumor, a routine immunohistochemistry antibody panel on the tumor cells revealed negative staining for estrogen receptor (ER), progesterone receptor (PR), and human epidermal growth factor receptor-2 (Her-2). Strong positive staining for the cluster of differentiation 10 (CD10) and vimentin was present.

**Conclusion:**

This case is unusual because of the site of metastatic progression. It is important for physicians to be aware of this progression so early diagnoses can be made, and appropriate therapeutic planning can be initiated.

## Background

Renal cell carcinoma (RCC) is a malignant tumor that often metastasizes early, and approximately 33% of RCC patients present with metastatic masses [1]. RCC commonly metastasizes to the lung parenchyma (45.2%), skeleton (29.5%), lymph nodes (20.8%), liver (20.3%), adrenals (8.9%) and brain (8.1%) [1]. Other locations of metastases from primary RCC are uncommon, and metastases to the breast from metastatic RCC are extremely rare. Compared with breast cancer, which is the most common primary malignancy in women, breast metastases from extramammary malignancies are quite uncommon. The rarity of RCC metastasis to the breast presents a unique challenge for accurately recognizing the metastatic disease. The clinical presentations of different metastatic sites are highly variable; however, malignancies in the breast often grows as solitary nodes without any obvious symptoms. These tumors are most commonly found incidentally on physical examinations. The metastatic spread of RCC to the breast is still unclear. Typically, most tumors metastasize through hematogenous or lymphatic routes. Since there is a paucity of literature on RCC metastasis to the breast, little is known about the patterns of spread and the association between metastatic RCC and its dissemination to the breast. Herein, we reported a case of RCC metastasis to the breast, described the tumor features identified on multiple breast imaging modalities, and discussed the previous reports of similar diseases.

## Case presentation

**Fig. 1:**
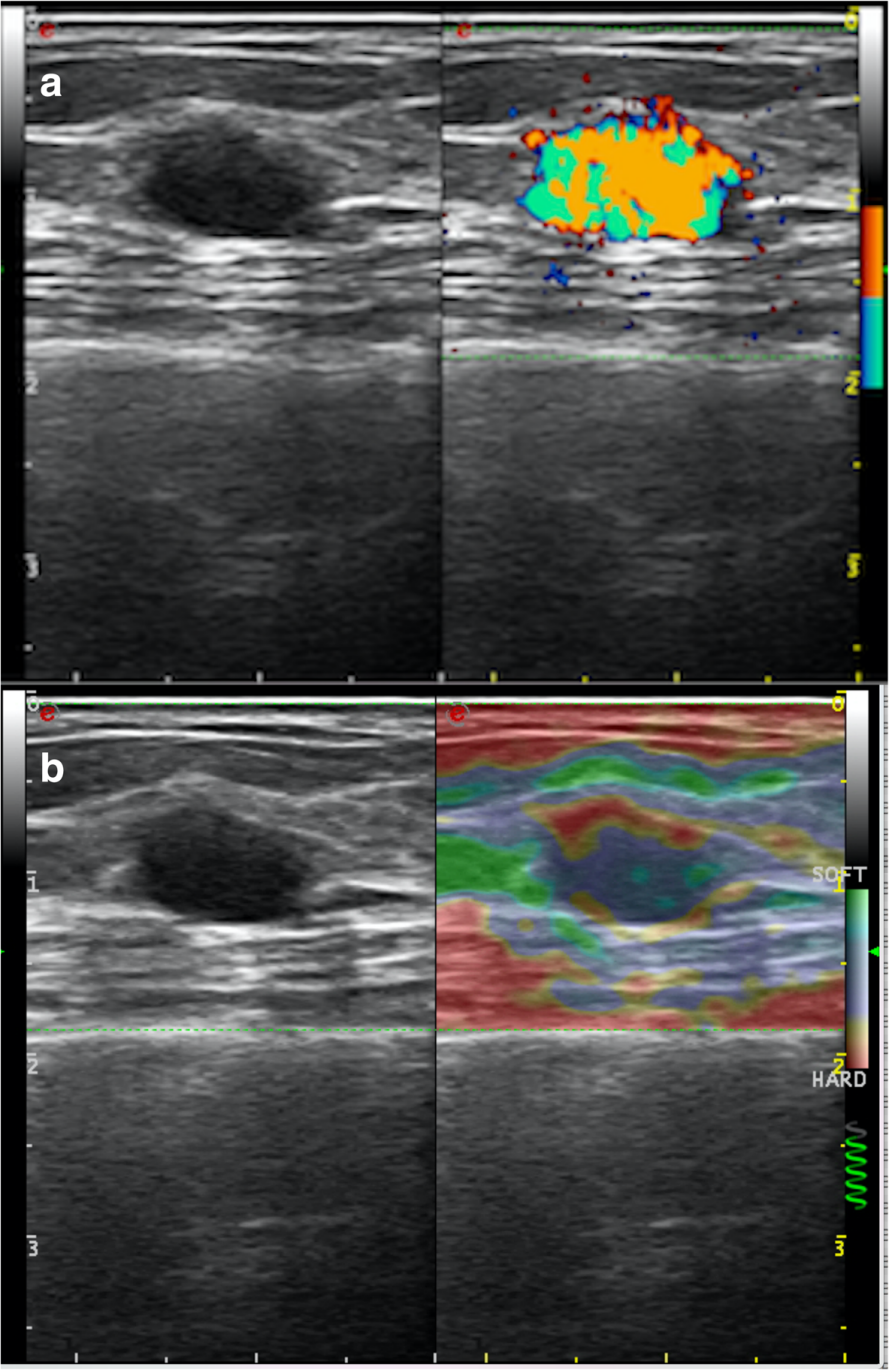
Breast ultrasonography. Ultrasound of the breast showed an oval, hypo-echoic nodule with well-defined margins and no shadowing. Extensive vascularity was also noted. The RI was 0.62–0.67 (**a**). Elastography revealed an E2 lesion (**b**)

**Fig. 2:**
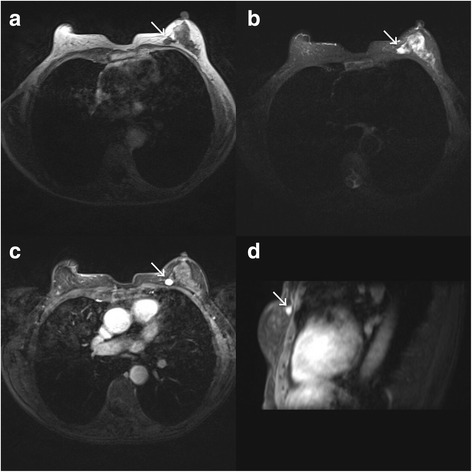
Breast MRI. Rounded lesion in the left breast was adjacent to the chest wall, hypo-intense on a T1-weighted image (**a**), hyperintense on a fat-saturated T2-weighted image (**b**) and was significantly enhanced following gadolinium administration (**c** and **d**)

A 68-year-old Chinese female was admitted to our department for an incidental discovery of a nodule in her left breast. She was diagnosed with grade 2 clear cell RCC 10 years earlier, and she underwent a nephrectomy for an incidental kidney mass found during a routine imaging examination. No abnormal findings were revealed during the physical examination or laboratory tests. Diagnostic mammography showed an asymmetric, dense shadow in the inner, upper quadrant of the left breast, and no obvious nodules were identified in the oblique view. Breast ultrasonography (US) of the left breast (Fig. 1) revealed a solid hypo-echoic nodule of approximately 10 mm × 6 mm in size with an oval shape, smooth edges and a well-defined boundary. Color Doppler flow imaging showed abnormal blood flow. The resistive index (RI) was 0.62–0.67. Elastography showed a completely soft lesion, which was classified as E2. We paid close attention to the extensive vascularity accompanying the nodule. Subsequent breast magnetic resonance imaging (MRI) (Fig. 2) revealed a nodule with marked homogenous contrast enhancement in the left breast adjacent to the chest wall. The nodule was given a score of IV B based on the Breast Imaging Reporting and Data System (BI-RADS) [2].

**Fig. 3:**
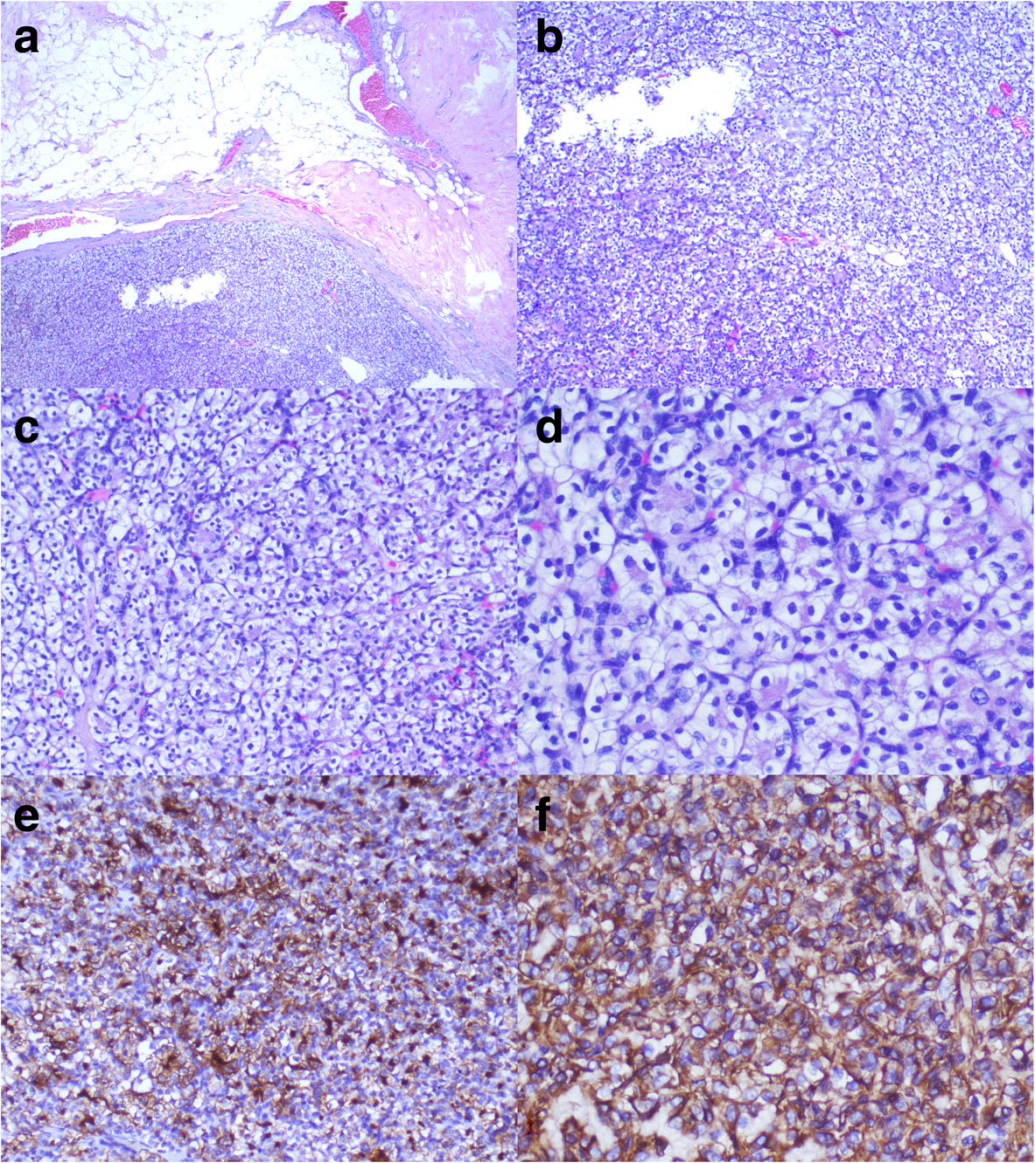
Renal clear cell carcinoma metastasis to the breast. **a** A low-power view showed a metastatic clear cell RCC in the breast with atrophic breast parenchyma in the background. The apparent circumscription and the pseudocapsule surrounding the carcinoma were noted (black arrows), (hematoxylin-eosin, original magnification 40×). **b** A medium-power view showed infiltration of clear cells into the surrounding breast tissue (hematoxylin-eosin, original magnification 100×). **c** Clear cells with distinct cytoplasmic borders were arranged in nests separated by a thin fibrovascular stroma (hematoxylin-eosin, original magnification 200×). **d** A high-power view revealed foamy, vacuolar cytoplasm and large nucleoli consistent with clear cell RCC (hematoxylin-eosin, original magnification 400×). **e** Renal neoplastic cell showing strong membranous staining with CD10 (immunohistochemistry, original magnification 400×). **f** Diffuse and strong cell cytoplasm staining for vimentin was observed in clear cell RCC. Positive cells displayed brownish yellow granules on the surface, cytoplasm, and intercalated discs (immunohistochemistry, original magnification 400×)

Due to the limited volume, deep location, and abundant vascularity of the lesion, as well as the risk of bleeding, percutaneous US-guided core needle biopsy was not performed. Surgical excision of the nodule was performed. Macroscopically, a suspicious nodule was found at the site of the specimen, but unfortunately, no pictures were taken during the surgery. Microscopically (Fig. 3a–d), the nodule was similar in appearance to the RCC in the primary renal site. Nests, sheets, and cords of polygonal cells were present in the nodule and were associated with a rich sinusoidal vascular network (Fig. 3a–d). Immunohistochemistry staining revealed strong positive labeling for the cluster of differentiation 10 (CD10) and vimentin, and an absence of labeling for estrogen receptor (ER), progesterone receptor (PR), and human epidermal growth factor receptor-2 (Her-2). This staining pattern further suggested metastatic RCC to the breast (Fig. 3e–f). Currently, there are no clinical or radiological findings regarding the recurrence of metastasis after 2 months of follow-up. The patient has undergone no further therapy since the surgery.

## Discussion and conclusions

RCC is a urological carcinoma that accounts for 3% of all adult malignancies, and is the most common renal malignancy that originates in the lining of the proximal convoluted tubules [3]. RCC commonly metastasizes to the lung parenchyma, bone, lymph nodes, liver, adrenals and brain [1]. RCC metastases to the breast have been rarely reported [4, 5]. According to Gravis et al. [5], metastatic RCC to the breast was observed in only two patients among a total of 558 metastatic RCC patients. We have presented a rare case of RCC metastasis to the breast with no clinical signs.

Breast metastases from extramammary malignancies are uncommon. The most common sources reported in the literature are lymphoma, leukemia and melanoma [6]. Other uncommon sources include carcinomas of the ovary, stomach and lung. According to Sung Hee Mun [6], metastasis to the breast may rarely occur from hypernephromas, carcinoid tumors, and as carcinomas of the liver, endometrium, tonsil, pancreas, perineum, pleura, bladder and cervix. According to previous studies [6, 7], metastatic diseases of the breast may include single or multiple lesions. These lesions are often round to oval shaped, well-circumscribed, hypo-echoic, non-spiculated, and calcified or architecturally distorted. These features are not specific for metastases as most benign tumors have similar features. These masses are commonly located in the superficial subcutaneous tissues or adjacent to the breast parenchyma. The lesions are also relatively rich in blood supply.

The images of the lesion in our case were hypo-echoic and showed rich intralesional vascularization. There was no calcification observed in the lesion, but it shared many features with metastatic breast disease, with the exception of abundant blood flow. Differentiating a metastatic lesion from a primary tumor (such as solid papillary carcinoma of the breast) or from a benign lesion is difficult. On radiographic examination, metastatic breast tumors are usually well-circumscribed without calcifications, while primary breast tumors usually have spiculations and/or microcalcifications and are always lobulated. Metastatic tumors do not involve the ducts and do not cause nipple retraction, skin dimpling, or nipple discharge [8, 9]. The lesion in our case was very similar to a benign nodule, except it had abundant blood flow. Most primary carcinomas of the breast have increased vascularity, but the lesion in this case showed much more vascularity than most primary breast carcinomas. Most cases of clear cell RCCs are hypervascular in nature, and according to Jinzaki et al. [10], clear cell RCC shows a high mean microvessel density of 653.6 mm^2^. Most primary breast carcinomas are stiff and non-elastic by nature with a score higher than E3, while the mass in this case appeared soft on elastography imaging with a score of E2. This could possibly be explained by the lesion composition: clear renal cells with foamy, vacuolar cytoplasm and large nucleoli. The cells had distinct cytoplasmic borders and were arranged in nests separated by thin fibrovascular stroma.

Although metastatic RCC and primary carcinoma of the breast share identical morphological features, there are other markers that can help to distinguish them. Histology and immunohistochemistry play important roles in the diagnosis of metastatic RCC. A high-power microscopic view revealed typical clear cell type, which included single cells with vacuolated cytoplasm, round nuclei, and prominent nucleoli. Normally, RCC cells are large with a low nuclear/cytoplasmic ratio [11]. These can be differentiated from other solid tumors, including breast metastases from extramammary malignancies such as adenomyoepithelioma, and from melanoma by negative immunostaining for myoepithelial (calponin), and melanocytic (HMB-45) markers. The tumor was surrounded by a pseudocapsule composed of fibrous tissue (Fig. 3a) which was useful for the differential diagnosis of RCC [12]. Pseudocapsules are uncommon in primary breast carcinoma and are useful for differentiating primary tumors from metastases. Immunohistochemistry revealed strong positive staining for CD10 (Fig. 3e) and vimentin (Fig. 3f), which was consistent with the diagnosis of RCC [13]. Our case was also negative for ER, PR and Her-2, as well as CK7 (−), CK20 (−), Villin (+), Ki-67 (3% +), and CK (+). Compared with metastatic RCC, tumor cells in primary solid papillary carcinoma of the breast are significantly associated with strong positive ER, PR and chromogranin A labeling, spindled tumor morphology, and advanced patient age [14]. Evaluating the characteristics of these lesions, in combination with patient medical histories and advanced patient age, might be helpful in diagnosing metastatic RCC lesions of the breast.

RCC can recur at any time after nephrectomy. The interval from diagnosis of a primary tumor to the detection of breast metastases varies from a few months to years. RCC recurrence occurs either in a synchronous or in a metachronous manner. Vassalli et al. [15] reported that in 14 cases, breast masses were found 1–18 years following nephrectomy for RCC. In 7 cases, the initial signs of metastatic disease of metastatic RCC to the breast were present at the time of nephrectomy. We reviewed all the related literature (Table 1), and to the best of our knowledge there is no strong evidence regarding a significant prognostic difference between metachronous and synchronous presentations. Overall, 32 cases have been reported in the literature. Twenty-five cases were presented in which metastasis occurred 3–18 years after nephrectomy for RCC, and 1 case was presented with evidence of bilateral, metachronous lesions after nephrectomy [8, 15–30]. Therefore, a history of RCC arouses suspicion of concerning breast metastasis, even though primary carcinoma of the breast is much more common. References not mentioned above are appended to the Table 1.

**Table 1:** Cases published in the literature on metastatic RCC to the breast

Year	Author	Age (yr)^a^	Pathologic type	Total cases	Time after Nephrectomy (yr)
1999	Chhieng DC [16]	39–78	renal cell adenocarcinoma	3	N/A
1999	Forte A [17]	N/A	RCC	1	many years
2001	Vassalli L [15]	N/A	RCC	14	1–18
2003	O’Sullivan AW [18]	N/A	RCC	1	N/A
2005	Gacci M [19]	79	clear cell RCC	1	3
2006	McLauglin AS [8]	76	clear cell RCC	1	12
2007	Hasteh F [20]	61	renal carcinoid tumor	1	5
2007	Alzaraa A [21]	81	RCC	1	5
2007	Lee WK [22]	72	RCC	1	N/A
2008	Daneshbod Y [23]^b^	65	clear cell RCC	1	8
2008	Ganapathi S [24]	88	RCC	1	4
2009	Durai R [25]	68	RCC	1	12
2011	Balliauw C [26]	N/A	RCC	1	N/A
2012	Pathe N [27]	N/A	RCC	1	N/A
2012	Mahrous M [28]	58	RCC	1	5
2013	Botticelli A [29]	60	clear cell RCC	1	4
2014	Falco G [30]	70	RCC	1	9

*RCC* renal cell carcinoma, *N/A* not available

^a^ Age during the year of diagnosis of breast metastasis

^b^ Bilateral breast metastases of renal carcinoma

The prognosis for patients presenting with metastatic RCC was dismal. The median survival time was only 6 to 12 months, and the 2-year survival rate was 10% to 20% [3]. Surgery played an important role in the management of patients with metastatic RCC. Recent, randomized, prospective trials suggested that there might be other viable treatment options, including cytoreductive nephrectomy, combined nephrectomy and metastasectomy, systemic immunotherapy, and biologic response modifier therapy [4]. As there are no specific management guidelines, and there is not an universal consensus for this extremely rare condition, metastatic RCC to the breast is treated similarly to other metastatic masses. Complete surgical resection of all suspected metastatic lesions might be the only option as the clinical management of patients with metastatic RCC is complicated due to lack of significant efficacy from available therapies [31]. Alternative treatment options might include cytoreductive nephrectomy, systemic immunotherapy and tumor embolization. Patient selection, performance status and disease burden should be considered when choosing a treatment [3]. Advances in biological response modifier therapy also brought new hope to some patients who responded positively to this therapy. Cytoreductive nephrectomy was taken into account as an integral part in the management of these patients [31].

In summary, we reported a case of metastatic RCC to the breast that mimicked some types of primary breast nodules. Although this case is unusual because of the site of metastatic progression, it is still important for physicians to be aware of such conditions to make early diagnoses and start appropriate therapeutic programs. Multiple breast imaging modalities, such as ultrasonography, can provide clues for differentiating metastatic RCC to the breast from benign breast masses. We also suggest a mandatory microhistological biopsy for new breast lesions in patients with a history of RCC.

## Abbreviations

CD10: Cluster of differentiation 10
ER: Estrogen receptor
Her-2: Human epidermal growth factor receptor
MRI: Magnetic resonance imaging
PR: Progesterone receptor
RCC: Renal cell carcinoma
RI: Resistive index
US: Ultrasonography

## References

[1] Bianchi M, Sun M, Jeldres C (2012). Distribution of metastatic sites in renal cell carcinoma: a population-based analysis. Ann of Oncol.

[2] American College of Radiology (ACR). ACR BI-RADS-magnetic resonance imaging / /ACR breast imaging reporting and data system, breast imaging atlas. Reston. 2003:17–95.

[3] Campbell SC, Flanigan RC, Clark JI (2003). Nephrectomy in metastatic renal cell carcinoma. Curr Treat Option On.

[4] Schlesinger-Raab A, Treiber U, Zaak D, Holzel D, Engel J (2008). Metastatic renal cell carcinoma: results of a population-based study with 25 years follow-up. Eur JCancer.

[5] Gravis G, Chanez B, Derosa L (2016). Effect of glandular metastases on overall survival of patients with metastatic clear cell renal cell carcinoma in the antiangiogenic therapy era. Urol Oncol.

[6] Mun SH, Ko EY, Han BK, Shin JH, Kim SJ, Cho EY (2014). Breast metastases from extramammary malignancies: typical and atypical ultrasound features. Korean J Radiol.

[7] Benveniste AP, Marom EM, Benveniste MF, Mawlawi OR, Miranda RN, Yang W (2014). Metastases to the breast from extramammary malignancies - PET/CT findings. EurJ Radiol.

[8] McLauglin SA, Thiel DD, Smith SL, Wehle MJ, Menke DM (2006). Solitary breast mass as initial presentation of clinically silent metastatic renal cell carcinoma. Breast.

[9] Lee AH (2007). The histological diagnosis of metastases to the breast from extramammary malignancies. J Clin Pathol.

[10] Jinzaki M, Tanimoto A, Mukai M (2000). Double-phase helical CT of small renal parenchymal neoplasms: correlation with pathologic findings and tumor angiogenesis. J Comput Assist Tomogr.

[11] Gettman MT, Blute ML, Spotts B, Bryant SC, Zincke H (2001). Pathologic staging of renal cell carcinoma: significance of tumor classification with the 1997 TNM staging system. Cancer.

[12] Jiang J, Chen Y, Zhou Y, Zhang H (2010). Clear cell renal cell carcinoma: contrast-enhanced ultrasound features relation to tumor size. Eur J Radiol.

[13] Truong LD, Shen SS (2011). Immunohistochemical diagnosis of renal neoplasms. Arch PatholLab Med.

[14] Tan BY, Thike AA, Ellis IO, Tan PH (2016). Clinicopathologic characteristics of solid papillary carcinoma of the breast. Am J Surg Pathol.

[15] Vassalli L, Ferrari VD, Simoncini E (2001). Solitary breast metastases from a renal cell carcinoma. Breast Cancer Res Treat.

[16] Chhieng DC, Cohen JM, Waisman J, Fernandez G, Skoog L, Cangiarella JF (1999). Fine-needle aspiration cytology of renal-cell adenocarcinoma metastatic to the breast: a report of three cases. Diagn Cytopathol.

[17] Forte A, Peronace MI, Gallinaro LS (1999). Metastasis to the breast of a renal carcinoma: a clinical case. Eur Rev Med Pharmacol Sci.

[18] O’Sullivan AW, Kelly PM, Smith JM, Gorey TF (2003). Renal cell carcinoma metastasis to breast. I J Med Sci.

[19] Gacci M, Orzalesi L, Distante V (2005). Renal cell carcinoma metastatic to the breast and breast cancer metastatic to the kidney: two rare solitary metastases. Breast J.

[20] Hasteh F, Pu R, Michael CW (2007). A metastatic renal carcinoid tumor presenting as breast mass: a diagnostic dilemma. Diagn Cytopatho.

[21] Alzaraa A, Vodovnik A, Montgomery H, Saeed M, Sharma N (2007). Breast metastasis from a renal cell cancer. World J SurgOncol.

[22] Lee WK, Cawson JN, Hill PA, Hoang J, Rouse H (2007). Renal cell carcinoma metastasis to the breast: mammographic, sonographic, CT, and pathologic correlation. Breast J.

[23] Daneshbod Y, Khojasteh HN, Atefi S, Aledavood A (2008). Renal cell carcinoma presenting as a solitary breast mass. A diagnostic pitfall on aspiration cytology of clear cell tumors of the breast. Breast J.

[24] Ganapathi S, Evans G, Hargest R (2008). Bilateral breast metastases of a renal carcinoma: a case report and review of the literature. BMJ Case Rep.

[25] Durai R, Ruhomauly SN, Wilson E, Hoque H (2009). Metastatic renal cell carcinoma presenting as a breast lump in a treated breast cancer patient. Singap Med J.

[26] Balliauw C, Termote B, Van Steen A, Moerman P, Christiaens MR, Van Ongeval C (2011). Metastatic renal cell carcinoma presenting as a breast mass in a woman with history of primary breast cancer. JBR-BTR.

[27] Pathe N, Raymond J, Cintra AU (2012). Metastatic renal cell cancer presenting as a breast mass. Cli Ad Hematol Oncol.

[28] Mahrous M, Al Morsy W, Al-Hujaily A, Al-Sulimani S (2012). Breast metastasis from renal cell carcinoma: rare initial presentation of disease recurrence after 5 years. J Breast Cancer.

[29] Botticelli A, De Francesco GP, Di Stefano D (2013). Breast metastasis from clear cell renal cell carcinoma. J Ultrasound.

[30] Falco G, Buggi F, Sanna PA, Dubini A, Folli S (2014). Breast metastases from a renal cell carcinoma. A case report and review of the literature. Int J Surg Case Rep.

[31] Flanigan RC, Campbell SC, Clark JI, Picken MM (2003). Metastatic renal cell carcinoma. Curr Treat Options in Oncol.

